# Supplementary material for: Guideline adherence in the management of head injury in Australian children: A population-based sample survey
Source: PLoS One. 2020 Feb 11;15(2):e0228715. doi: 10.1371/journal.pone.0228715 (PMC7012413; doi:10.1371/journal.pone.0228715)
Supplement: S1 Table — ID = Identifier; GP = General Practitioner; ED = Emergency Department; IP = Inpatient; GCS = Glasgow Coma Scale; AVPU = Alert/Pain/Voice/Unresponsive; CSF = Cerebrospinal Fluid; AV = arteriovenous; CO2 = Carbon dioxide; PaO2 = Partial pressure of oxygen; PaCO2 = Partial pressure of carbon dioxide; SaO2 = Arterial oxygen saturation; IV = Intravenous; IM = Intra-muscular; IN = Intra-nasal; ED = Emergency Department. # Strength of recommendation as reported in individual CPGs. CPGs used a variety of classification schemes for allocating strength of recommendation in Grades (with A indicating the strongest recommendation in all classification schemes). If Grades were not specified in the CPG, or a Level of Evidence category, the term “Consensus-based recommendation” was assigned. * The type of quality of care assessed was classified as underuse or overuse: underuse refers to actions which are recommended, but not undertaken; overuse refers to actions which are not indicated or contraindicated in the context of the indicator’s inclusion criteria. (DOCX) [file pone.0228715.s001.docx]

**S1 Table 1: Characteristics, by clinical indicator, 2012 - 2013**

|  | | | **No. of Sites** | | |  | | |
| --- | --- | --- | --- | --- | --- | --- | --- | --- |
| **Indicator ID** | **Indicator Description** | **Age Inclusion Criteria** | **GP** | **ED** | **IP** | **Strength of Recommendation^#^** | **Phase of Care** | **Quality Type*** |
| HEAD01 | Children who presented with a head injury and any of the following: * unconscious/responding only to pain OR * fitting OR * signs of cardiovascular compromise were categorised as a Triage 1 patient. | 0 - 15 years | NA | 7 | 3 | Consensus-based recommendation | Diagnosis | Underuse |
| HEAD02 | Children who presented with a head injury and any of the following: * abnormal drowsiness/ responding only to voice OR * loss of consciousness of more than 5 minutes OR * focal signs OR * severe pain or headache OR * high risk mechanism were categorised as a Triage 2 patient. | 0 - 15 years | NA | 26 | 7 | Consensus-based recommendation | Diagnosis | Underuse |
| HEAD03 | Children who presented with a head injury and any of the following: * alert but altered behaviour OR * loss of consciousness less than 5 minutes OR * moderate pain or headache OR * moderate risk mechanism OR * significant neurological, developmental or bleeding comorbidities OR * less than one year of age OR * possible inflicted head injury, otherwise well were categorised as a Triage 3 patient. | 0 - 15 years | NA | 34 | 14 | Consensus-based recommendation | Diagnosis | Underuse |
| HEAD04 | Children aged ≥ 12 months who presented with an acute head injury and ONLY the following features: * low impact mechanism AND * NO neurological signs or symptoms AND * NO comorbidities or concerns regarding inflicted head injury were categorised as a Triage 4 or 5 patient. | 1 - 15 years | NA | 31 | 2 | Consensus-based recommendation | Diagnosis | Underuse |
| HEAD05 | Children who presented with a moderate to severe head injury (GCS 3-13) received a primary survey and assessment of their airway (with cervical spine immobilisation). | 0 - 15 years | 2 | 20 | 9 | Consensus-based recommendation | Treatment | Underuse |
| HEAD06 | Children who presented with a moderate to severe head injury (GCS 3-13) received a primary survey and assessment of their breathing function. | 0 - 15 years | 2 | 20 | 9 | Consensus-based recommendation | Treatment | Underuse |
| HEAD07 | Children who presented with a moderate to severe head injury (GCS 3-13) received a primary survey and assessment of their circulation. | 0 - 15 years | 2 | 20 | 9 | Consensus-based recommendation | Treatment | Underuse |
| HEAD08 | Children who presented with a moderate to severe head injury (GCS 3-13) received a primary survey and assessment of their pupil size and reaction to light. | 0 - 15 years | 2 | 20 | 9 | Consensus-based recommendation | Treatment | Underuse |
| HEAD09 | Children who presented with a moderate to severe head injury (GCS 3-13) received a primary survey and assessment of their GCS or AVPU. | 0 - 15 years | 2 | 20 | 9 | Consensus-based recommendation | Treatment | Underuse |
| HEAD10 | Children who presented with a moderate to severe head injury (GCS 3-13) received a primary survey and assessment of their blood glucose. | 0 - 15 years | 2 | 20 | 9 | Consensus-based recommendation | Treatment | Underuse |
| HEAD11 | Children who presented with a moderate to severe head injury (GCS 3-13) received a secondary survey which included palpation for bogginess, swelling or bruising of the scalp. | 0 - 15 years | 2 | 20 | 10 | Consensus-based recommendation | Treatment | Underuse |
| HEAD12 | Children who presented with a moderate to severe head injury (GCS 3-13) received a secondary survey which included looking for signs of base of skull fracture such as Battle's sign (bruising over mastoid), 'raccoon' eyes or blood behind the ear drum. | 0 - 15 years | 2 | 20 | 10 | Consensus-based recommendation | Treatment | Underuse |
| HEAD13 | Children who presented with a moderate to severe head injury (GCS 3-13) received a secondary survey which included examination for haemo-tympanum or signs of CSF leak from ears or nose. | 0 - 15 years | 2 | 20 | 10 | Consensus-based recommendation | Treatment | Underuse |
| HEAD14 | Children who presented with a moderate to severe head injury (GCS 3-13) received a secondary survey which included an examination for facial (e.g. nose, mouth, ears) deformities, swelling, bleeding, lacerations, tenderness. | 0 - 15 years | 2 | 20 | 10 | Consensus-based recommendation | Treatment | Underuse |
| HEAD15 | Children who presented with a moderate to severe head injury (GCS 3-13) received a secondary survey which included examination for cervical spine deformity, tenderness, muscle spasm, crepitus, motor function, reflexes and lateralising signs. | 0 - 15 years | 2 | 20 | 10 | Consensus-based recommendation | Treatment | Underuse |
| HEAD16 | Children who presented with a head injury had their history documented which included the time of injury. | 0 - 15 years | 53 | 34 | 25 | Consensus-based recommendation | Treatment | Underuse |
| HEAD17 | Children who presented with a head injury had their history documented which included mechanism of injury. | 0 - 15 years | 53 | 34 | 25 | Consensus-based recommendation | Treatment | Underuse |
| HEAD18 | Children who presented with a head injury had their history documented which included a recall of events. | 0 - 15 years | 51 | 34 | 25 | Consensus-based recommendation | Treatment | Underuse |
| HEAD19 | Children who presented with a head injury had their history documented which included whether there was loss or impairment of consciousness (and duration). | 0 - 15 years | 53 | 34 | 25 | Consensus-based recommendation | Treatment | Underuse |
| HEAD20 | Children who presented with a head injury had their history documented which included the presence/absence of seizures. | 0 - 15 years | 53 | 34 | 25 | Consensus-based recommendation | Treatment | Underuse |
| HEAD21 | Children who presented with a head injury had their history documented which included their behaviour and activity since the time of injury. | 0 - 15 years | 53 | 34 | 25 | Consensus-based recommendation | Treatment | Underuse |
| HEAD22 | Children who presented with a head injury had their history documented which included whether they had any nausea or vomiting. | 0 - 15 years | 53 | 34 | 25 | Consensus-based recommendation | Treatment | Underuse |
| HEAD23 | Children who presented with a head injury had their history documented which included their clinical course prior to consultation, e.g. stable, deteriorating, improving. | 0 - 15 years | 53 | 34 | 25 | Consensus-based recommendation | Treatment | Underuse |
| HEAD24 | Children who presented with a head injury had their history documented which included any other injuries sustained. | 0 - 15 years | 53 | 34 | 25 | Consensus-based recommendation | Treatment | Underuse |
| HEAD25 | Children who presented with a head injury had their history documented which included comorbidities that predispose to intracranial injury (intra-cerebral shunt, AV malformation, bleeding disorders (including vitamin K deficiency). | 0 - 15 years | 32 | 28 | 19 | Consensus-based recommendation | Treatment | Underuse |
| HEAD26 | Children who presented to the ED with a head injury and any of the following: * GCS persistently less than or equal to 8 OR * loss of protective laryngeal reflexes OR * abnormal breathing pattern or hypoventilation OR * oxygen saturation less than or equal to SpO₂ 95% or a PaO₂ less than 80 mmHg on maximal facial oxygen OR * PaCO₂ less than 30 mmHg or PaCO₂ greater than 44 mmHg were classified as severe and were intubated and ventilated. | 0 - 15 years | NA | 9 | NA | Consensus-based recommendation | Treatment | Underuse |
| HEAD27 | Children with a severe head injury (GCS 3-8) received immobilisation of their cervical spine. | 0 - 15 years | NA | 9 | 6 | Consensus-based recommendation | Treatment | Underuse |
| HEAD28 | Children with a severe head injury (GCS 3-8) who had completed their fluid resuscitation, were nursed 20-30 degrees head up. | 0 - 15 years | NA | 5 | 5 | Consensus-based recommendation | Treatment | Underuse |
| HEAD29 | Children with a severe head injury (GCS 3-8) received continuous cardio-respiratory (respiratory rate, pulse) and oxygen saturation monitoring. | 0 - 15 years | NA | 9 | 5 | Consensus-based recommendation | Treatment | Underuse |
| HEAD30 | Children with a severe head injury (GCS 3-8) had their BP measured every 15-30 minutes. | 0 - 15 years | NA | 9 | 5 | Consensus-based recommendation | Treatment | Underuse |
| HEAD31 | Children with a severe head injury (GCS 3-8) who were not intubated, had their GCS recorded every 15-30 minutes. | 0 - 15 years | NA | 5 | 3 | Consensus-based recommendation | Treatment | Underuse |
| HEAD32 | Children with a severe head injury (GCS 3-8) received an urgent CT of the head. | 0 - 15 years | NA | 8 | 5 | Grade B | Treatment | Underuse |
| HEAD33 | Children with a severe head injury (GCS 3-8) received an urgent C-Spine CT. | 0 - 15 years | NA | 8 | 5 | Consensus-based recommendation | Treatment | Underuse |
| HEAD34 | Children with a severe head injury (GCS 3-8) received a consultation with ICU and neurosurgical specialists. | 0 - 15 years | NA | 8 | 5 | Consensus-based recommendation | Treatment | Underuse |
| HEAD35 | Children who presented with moderate head injury (GCS 9-13) without neurological deterioration had their GCS observed in hospital at least half-hourly for a minimum of four hours. | 0 - 15 years | NA | 13 | 1 | Consensus-based recommendation | Treatment | Underuse |
| HEAD36 | Children who presented with moderate head injury (GCS 9-13) without neurological deterioration had their pulse rate observed in hospital at least half-hourly for a minimum of four hours. | 0 - 15 years | NA | 14 | 2 | Consensus-based recommendation | Treatment | Underuse |
| HEAD37 | Children who presented with moderate head injury (GCS 9-13) without neurological deterioration had their respiratory rate observed in hospital at least half-hourly for a minimum of four hours. | 0 - 15 years | NA | 14 | 2 | Consensus-based recommendation | Treatment | Underuse |
| HEAD38 | Children who presented with moderate head injury (GCS 9-13) without neurological deterioration had their blood pressure observed in hospital at least half-hourly for a minimum of four hours. | 0 - 15 years | NA | 14 | 2 | Consensus-based recommendation | Treatment | Underuse |
| HEAD39 | Children who presented with moderate head injury (GCS 9-13) without neurological deterioration had their pupils assessed in hospital at least half-hourly for a minimum of four hours. | 0 - 15 years | NA | 14 | 2 | Consensus-based recommendation | Treatment | Underuse |
| HEAD40 | Children who presented with moderate head injury (GCS 9-13) without neurological deterioration had their limb strength assessed in hospital at least half-hourly for a minimum of four hours. | 0 - 15 years | NA | 14 | 2 | Consensus-based recommendation | Treatment | Underuse |
| HEAD41 | Children with a moderate/intermediate head injury (GCS 9-13) who experienced an acute deterioration including persistent vomiting (at 6 hours post injury) received a CT of the head. | 0 - 15 years | NA | 6 | 1 | Grade B | Treatment | Underuse |
| HEAD42 | Children with a moderate/intermediate head injury (GCS 9-13) who experienced an acute deterioration including persistent headache (at 6 hours post injury) received a CT of the head. | 0 - 15 years | NA | 4 | 0 | Grade B | Treatment | Underuse |
| HEAD43 | Children with a moderate/intermediate head injury (GCS 9-13) who experienced an acute deterioration including persistent irritability (at 6 hours post injury) received a CT of the head. | 0 - 15 years | NA | 4 | 1 | Grade B | Treatment | Underuse |
| HEAD44 | Children with a moderate/intermediate head injury (GCS 9-13) who experienced an acute deterioration including persistent abnormal behaviour/neurological abnormality (at 6 hours post injury) received a CT of the head. | 0 - 15 years | NA | 6 | 1 | Grade B | Treatment | Underuse |
| HEAD45 | Children with a moderate/intermediate head injury (GCS 9-13) who experienced an acute deterioration including persistent unsteady gait (at 6 hours post injury) received a CT of the head. | 0 - 15 years | NA | 1 | 0 | Grade B | Treatment | Underuse |
| HEAD46 | Children who presented with a head injury were not intubated via a nasotracheal airway. | 0 - 15 years | NA | 34 | 25 | Consensus-based recommendation | Treatment | Overuse |
| HEAD47 | Children who presented with a head injury did not receive a nasogastric tube. | 0 - 15 years | NA | 34 | 25 | Consensus-based recommendation | Treatment | Overuse |
| HEAD48 | Children with a head injury who were intubated had end tidal CO₂ monitoring. | 0 - 15 years | NA | 15 | 7 | Consensus-based recommendation | Treatment | Underuse |
| HEAD49 | Children with a head injury who were intubated had PaO₂ greater than 80 mmHg (SaO₂ greater than 95%). | 0 - 15 years | NA | 12 | 5 | Consensus-based recommendation | Treatment | Underuse |
| HEAD50 | Children with a head injury who were intubated had PaCO₂ between 35-40 mmHg. | 0 - 15 years | NA | 12 | 5 | Consensus-based recommendation | Treatment | Underuse |
| HEAD51 | Children who presented with a head injury and any of the following: * GCS less than 15 OR * posterior bony neck pain or tenderness OR * focal deficit at any time since injury OR * paraethesia in the extremities OR * distracting injury OR * intoxication received cervical spine precautions. | 0 - 15 years | NA | 24 | 9 | Consensus-based recommendation | Treatment | Underuse |
| HEAD52 | Children who presented with head injury who were seizing, were immediately administered: * midazolam (0.15 mg/kg bolus IV), OR * diazepam (0.25 mg/kg bolus IV) OR * midazolam 0.15 mg/kg IM, 0.5 mg/kg IN or 0.5 mg/kg buccal. | 0 - 15 years | NA | 4 | 1 | Consensus-based recommendation | Treatment | Underuse |
| HEAD53 | Children who presented with head injury and received sedation and/or opioid analgesia had their GCS recorded every 15 minutes until their GCS returned to the pre-sedation level. | 0 - 15 years | NA | 13 | 9 | Consensus-based recommendation | Treatment | Underuse |
| HEAD54 | Children with a minor/mild head injury (GCS 14-15) whose parents were provided with information on when to return to the ED if deterioration occurs, were discharged from the ED without a period of observation. | 0 - 15 years | NA | 33 | NA | Consensus-based recommendation | Ongoing management | Underuse |

**Legend:** ID=Identifier; GP=General Practitioner; ED=Emergency Department; IP=Inpatient; GCS=Glasgow Coma Scale; AVPU=Alert/Pain/Voice/Unresponsive; CSF=Cerebrospinal Fluid; AV=arteriovenous; CO₂=Carbon dioxide; PaO_2_=Partial pressure of oxygen; PaCO_2_=Partial pressure of carbon dioxide; SaO_2_=Arterial oxygen saturation; IV=Intravenous; IM=Intra-muscular; IN=Intra-nasal; ED=Emergency Department.

# Strength of recommendation as reported in individual CPGs. CPGs used a variety of classification schemes for allocating strength of recommendation in Grades (with A indicating the strongest recommendation in all classification schemes). If Grades were not specified in the CPG, or a Level of Evidence category, the term “Consensus-based recommendation” was assigned.

* The type of quality of care assessed was classified as underuse or overuse: underuse refers to actions which are recommended, but not undertaken; overuse refers to actions which are not indicated or contraindicated in the context of the indicator’s inclusion criteria.
