# Supplementary material for: Guideline adherence in the management of head injury in Australian children: A population-based sample survey
Source: PLoS One. 2020 Feb 11;15(2):e0228715. doi: 10.1371/journal.pone.0228715 (PMC7012413; doi:10.1371/journal.pone.0228715)
Supplement: S1 File — (DOCX) [file pone.0228715.s002.docx]

# S2: Additional details relating to study methods

The report of top-level CareTrack Kids (CTK) results[1] and its associated online appendix, detail the methods of the larger study, which generated the data reported in this paper. Selected methods specifically relevant to head injury are described below.

**Sample size**

A visit was defined as an occasion of admitted inpatient care, an Emergency Department (ED) presentation or a consultation with a General Practitioner (GP). Without adjustment for the design effect, a minimum of 400 visits per condition was required to obtain national estimates with 95% Confidence Interval (CI) and precision of +/- 5% at condition level, conservatively assuming only one eligible indicator per visit. It was anticipated that loss of precision due to design effects would be largely offset by multiple eligible indicators per visit and additional visits identified by the secondary sampling (multiple visits for care of head injury for each medical record identified for sampling of head injury, and visits for care of head injury incidentally found in medical records identified for sampling other conditions).

**Sampling** **Process**

A multistage stratified random sampling process was implemented. For logistical efficiency, sampling was targeted at three states, Queensland (QLD), New South Wales (NSW) and South Australia (SA), which together comprise 60.0% of the estimated Australian population aged 15 years or younger in the 2012 and 2013 calendar years. All six paediatric tertiary hospitals (two in QLD, three in NSW, and one in SA) were targeted as they have state-wide coverage. State Departments of Health organize care within administrative units (‘health districts’): Hospital Health Services in QLD, Local Health Districts in NSW, and Local Health Networks in SA. For QLD, we targeted five health districts (two metropolitan, three regional), in NSW four health districts (two metropolitan, two regional), and in SA three health districts (two metropolitan, one regional).

**Recruitment of health care providers**

Within the selected health districts, we approached all public hospitals, or private hospitals providing public services under contract, that had patient volumes of ≥2,000 ED presentations and ≥500 paediatric separations per year; we also advertised the study to GPs and approached all the providers we could identify through internet searches, and via personal contacts. Within the selected sites, we sampled medical records for each condition targeted at that setting.

Recruitment of GPs was decentralized. Administrative details for refusal rates, from cold-calling or direct contact by clinicians who facilitated recruitment of their peers, were maintained on project laptops. At the end of recruitment all computers were decommissioned and cleaned, with the files archived on a USB drive. Unfortunately, the USB drives created during laptop decommissioning were misplaced and have not been able to be located. This did not affect the indicator adherence data, as the database was remotely located and updated regularly via the internet. We have therefore sought to estimate the recruitment rates based on recruitment spreadsheets emailed to the administrative staff.

For GPs, we were only able to locate emailed spreadsheets with late stage records for one state, South Australia. Based on this spreadsheet, we approached 114 GPs and recruited 27 of them, giving a recruitment rate of 23.7%; an additional GP, not listed on the available spreadsheet, was recruited subsequently and was not added to either the numerator or the denominator, for this estimate. The spreadsheet did not have clear information on eligibility, so it is likely that an unknown number of the 114 approached were ineligible because: 1) they were not open during the whole 2012-2013 survey period; 2) they saw no or few children; or 3) they were not confident in their ability to generate full listings of children with the target conditions, or they did not use one of the four practice software systems our surveyors were trained to search. Our estimate of 23.7% is therefore likely to be an underestimate of the actual recruitment rate.

Self-selection of GPs could lead to bias in the estimated guideline adherence. It is plausible that self-selected practices were more confident of their guideline adherence, potentially leading to overestimation of guideline-adherence in the CareTrack Kids study.

**Allocation of visits to sampling units**

The number of head injury records targeted at each site was determined by a nominal allocation of the 400 records targeted, informed by data available at the time, supplemented by expert opinion, with planned over-sampling of settings where fewer occasions of care were expected.[1, 2] For hospitals, a fixed number was targeted at each site; for GPs, different combinations of conditions were targeted at each site, to simplify the logistics of sampling.

**Data collection**

Nine experienced paediatric nurses were employed across the three states, with all nine assessing occasions of care for head injury. The surveyors undertook a one-week training program, prior to data collection. A surveyor manual was developed which included instructions, condition-specific definitions, inclusion and exclusion criteria, and guidance for assessing eligibility of each encounter for relevant indicators. Mock records were assessed during the surveying task for 6 of the 9 surveyors (2 had already terminated employment and 1 was excluded as their assessments may not have been made independently) and their results compared. A good level of agreement was found; κ = 0.76 (95%CI, 0.75-0.77; n = 1895) for the child’s eligibility for indicator assessment, and κ = 0.71 (95% CI, 0.69-0.73; n = 1009) for indicator assessment.[1]

A web-based tool, originally developed for the CareTrack Adults study[3, 4], was designed to enter data during medical record review. Algorithms to filter indicators by healthcare setting, and by age, were embedded in the tool. One age-specific and a number of setting-specific filters were in place for head injury: for example, HEAD27, which assesses whether “Children with a severe head injury (Glasgow Coma Scale [GSC] score of 3-8) received immobilisation of their cervical spine”, was only asked for ED and inpatient visits.

Surveyors undertook criterion-based medical record reviews using the data collection tool. Medical records for selected visits in 2012 and 2013 were reviewed on-site at each participating facility during March–October 2016. The surveyors responded to each indicator as ‘Yes’ (care provided during the encounter was consistent with the indicator), ‘No’, or ‘Not Applicable’ (NA; the indicator was not eligible for assessment). For example, a surveyor assessing an occasion of care for a child with a GCS score <3 or >8, would record ‘NA’ to indicator HEAD27.

**Analysis**

Survey or register-derived data were used to estimate the proportion of occasions of care for head injury in each setting.[5-10] The number of occasions of healthcare for each condition was thereby estimated for each hospital or, for GPs, each health district, and sampling weights were calculated using the methods detailed in eAppendix 4 of the report of the top-line CTK results (this Appendix can be accessed by request via the corresponding author, if required).[1]

A variety of stratifications, and sometimes domain analysis,[11, 12] were necessary to ensure accuracy of the confidence interval estimates. These are detailed in eTable 1, below. Clustering was adjusted for by identifying the primary clustering unit, the health district; controlling for the primary clustering unit adjusts the confidence intervals for cluster-related variation for all stages of clustering below the primary unit.[12]

**eTable 1: Domain analysis and stratifications for different estimates presented in the manuscript.**

| Location | Sub-section/Area | Domain analysis[11, 12] | Strata |
| --- | --- | --- | --- |
| Table 2 | Indicator estimates | Yes | State and healthcare setting |
| Table 3 | Bundle A-B x healthcare setting estimates | Yes | None |
|  | Bundle A-B – Overall estimates | Yes | State and healthcare setting |
|  | Sub-bundle A1-A2 x healthcare setting estimates | Yes | None |
|  | Sub-bundle A1-A2 – Overall estimates | Yes | State and healthcare setting^#^ |
| Table 4 | Metro x healthcare setting estimates | Yes | State |
|  | Geographic Regions and Tertiary hospitals x healthcare setting estimates | Yes | None |

## ^#^ In QLD, healthcare setting was analysed using pseudo-strata with ED and GP aggregated, to avoid underestimation of variance due to single cluster for GP.

## References:

1. Braithwaite J, Hibbert PD, Jaffe A, et al. Quality of health care for children in Australia, 2012-2013. JAMA. 2018;319(11):1113-24.

2. Hooper TD, Hibbert PD, Mealing N, Wiles LK, Jaffe A, White L, et al. CareTrack Kids-part 2. Assessing the appropriateness of the healthcare delivered to Australian children: study protocol for a retrospective medical record review. BMJ Open. 2015;5(4):e007749.

3. Hunt TD, Ramanathan SA, Hannaford NA, Hibbert PD, Braithwaite J, Coiera E, et al. CareTrack Australia: assessing the appropriateness of adult healthcare: protocol for a retrospective medical record review. BMJ Open. 2012;2(1):e000665.

4. Runciman WB, Hunt TD, Hannaford NA, Hibbert PD, Westbrook JI, Coiera EW, et al. CareTrack: assessing the appropriateness of health care delivery in Australia. Med J Aust. 2012;197(2):100-5.

5. Britt H, Miller GC, Henderson J, Bayram C, Valenti L, Harrison C, et al. General Practice Activity in Australia 2012-13: BEACH: Bettering the Evaluation and Care of Health: Sydney University Press; 2013.

6. Harrison C. BEACH 2012-13 weighted data on frequency of management of selected conditions, for children aged 0-15, by General Practitioners. [Personal communication] Menzies Centre for Health Policy, School of Public Health, The University of Sydney; 2017.

7. Australian Institute of Health and Welfare. Australian hospital statistics 2012–13: Emergency department care. Canberra, AU: AIHW; 2013.

8. Queensland Health, New South Wales Health, South Australian Department of Health. Emergency Department data on frequency of management of selected conditions, for children aged 0-15. [Personal communication] 2017.

9. Australian Institute of Health and Welfare. Australian hospital statistics 2012–13. Canberra, AU: AIHW; 2014.

10. Australian Institute of Health and Welfare. Inpatient separations for selected conditions, as identified by ICD-10 principal diagnoses 2017. Available from: <http://www.aihw.gov.au/hospitals-data/principal-diagnosis-data-cubes/>.

11. Lohr S. Sampling: design and analysis. Second ed. Boston, MA: Brooks/Cole; 2009.

12. Heeringa SG, West BT, Berglund PA. Applied survey data analysis. Boca Raton, FL: CRC Press; 2010.
