# Supplementary material for: Guideline adherence in the management of head injury in Australian children: A population-based sample survey
Source: PLoS One. 2020 Feb 11;15(2):e0228715. doi: 10.1371/journal.pone.0228715 (PMC7012413; doi:10.1371/journal.pone.0228715)
Supplement: Attachment — Submitted filename: Response to Reviewers.docx [file pone.0228715.s003.docx]

| PONE-D-19-26245 entitled "Guideline Adherence in the Management of Head Injury in Australian Children: A Population-Based Sample Survey.” | |
| --- | --- |
| Itamar Ashkenazi  Academic Editor, PLOS ONE [general instructions] | |
| Thank you for submitting your manuscript to PLOS ONE. After careful consideration, we feel that it has merit but does not fully meet PLOS ONE’s publication criteria as it currently stands. Therefore, we invite you to submit a revised version of the manuscript that addresses the points raised during the review process.  We would appreciate receiving your revised manuscript by **Dec 12 2019 11:59PM**. When you are ready to submit your revision, log on to https://www.editorialmanager.com/pone/ and select the 'Submissions Needing Revision' folder to locate your manuscript file.  If you would like to make changes to your financial disclosure, please include your updated statement in your cover letter.  To enhance the reproducibility of your results, we recommend that if applicable you deposit your laboratory protocols in protocols.io, where a protocol can be assigned its own identifier (DOI) such that it can be cited independently in the future. For instructions see: http://journals.plos.org/plosone/s/submission-guidelines#loc-laboratory-protocols  Please include the following items when submitting your revised manuscript:   - A rebuttal letter that responds to each point raised by the academic editor and reviewer(s). This letter should be uploaded as separate file and labeled 'Response to Reviewers'. - A marked-up copy of your manuscript that highlights changes made to the original version. This file should be uploaded as separate file and labeled 'Revised Manuscript with Track Changes'. - An unmarked version of your revised paper without tracked changes. This file should be uploaded as separate file and labeled 'Manuscript'.   Please note while forming your response, if your article is accepted, you may have the opportunity to make the peer review history publicly available. The record will include editor decision letters (with reviews) and your responses to reviewer comments. If eligible, we will contact you to opt in or out.  We look forward to receiving your revised manuscript.  Kind regards,  Itamar Ashkenazi  Academic Editor  PLOS ONE | Thank you. Please find an itemised response below to all the comments. |
| 1. When submitting your revision, we need you to address these additional requirements. Please ensure that your manuscript meets PLOS ONE's style requirements, including those for file naming. The PLOS ONE style templates can be found at [http://www.journals.plos.org/plosone/s/file?id=wjVg/PLOSOne_formatting_sample_main_body.pdf](https://protect-au.mimecast.com/s/DEYnCnx1Z5UKw6K9UNTDZ7?domain=journals.plos.org) and [http://www.journals.plos.org/plosone/s/file?id=ba62/PLOSOne_formatting_sample_title_authors_affiliations.pdf](https://protect-au.mimecast.com/s/prhtCoV1Y2SB9KB2h6c1fi?domain=journals.plos.org) | This has been done. |
| 2.  Please include captions for your Supporting Information files at the end of your manuscript, and update any in-text citations to match accordingly. Please see our Supporting Information guidelines for more information: [http://journals.plos.org/plosone/s/supporting-information](https://protect-au.mimecast.com/s/Bf3GCp81gYCvkxv4hJkSuV?domain=journals.plos.org) | This has been done. |
| 3. We note that you have indicated that data from this study are available upon request. PLOS only allows data to be available upon request if there are legal or ethical restrictions on sharing data publicly. For information on unacceptable data access restrictions, please see [http://journals.plos.org/plosone/s/data-availability#loc-unacceptable-data-access-restrictions](https://protect-au.mimecast.com/s/b0F-Cq71jxfzB7zYirTfR7?domain=journals.plos.org). | A full statement of the Ethics requirements around limited data sharing are given. Given the number of bodies that must grant approval for data within their jurisdiction, we direct requests for access to the CTK Data Manager via email. We have amended this in the Declarations section. |
| In your revised cover letter, please address the following prompts: a) If there are ethical or legal restrictions on sharing a de-identified data set, please explain them in detail (e.g., data contain potentially identifying or sensitive patient information) and who has imposed them (e.g., an ethics committee). Please also provide contact information for a data access committee, ethics committee, or other institutional body to which data requests may be sent. b) If there are no restrictions, please upload the minimal anonymized data set necessary to replicate your study findings as either Supporting Information files or to a stable, public repository and provide us with the relevant URLs, DOIs, or accession numbers. Please see [http://www.bmj.com/content/340/bmj.c181.long](https://protect-au.mimecast.com/s/ZRbkCr810kC1MD1QtGva8M?domain=bmj.com) for guidelines on how to de-identify and prepare clinical data for publication. For a list of acceptable repositories, please see [http://journals.plos.org/plosone/s/data-availability#loc-recommended-repositories](https://protect-au.mimecast.com/s/2Ar3Cvl1g2S21y2wF84pAQ?domain=journals.plos.org). We will update your Data Availability statement on your behalf to reflect the information you provide. |  |
| *REVIEWER COMMENTS TO AUTHOR: Itamar Ashkenazi | |
| 1. Is the manuscript technically sound, and do the data support the conclusions?  Reviewer #1: Partly | See below |
| 2. Has the statistical analysis been performed appropriately and rigorously?  Reviewer #1: N/A |  |
| 3. Have the authors made all data underlying the findings in their manuscript fully available? Reviewer #1: Yes |  |
| 4. Is the manuscript presented in an intelligible fashion and written in standard English? Reviewer #1: Yes |  |
| 5. Review Comments to the Author Reviewer #1: Manuscript: Guideline adherence in the management of head injury in Australian Children: a population-based sample survey (PONE-D-19-26245)  Study type: survey of quality indicators  Authors' methodology and main findings: The authors compiled a list of quality indicators based upon guidelines and refined by a Delphi process. General practitioners' (27 of 114 approached), emergency department records, and hospitalized patient records of 629 children injured between 2012-2013 were evaluated. The authors found that adherence to avoidance of nasotracheal airways and nasogastric tubes was high, while documentation of past history and presence/absence of seizures was low.  Reviewer's comments:  This article deals with an important issue of indicators in treatment of head injury. While the findings of this study are not generalizable, the method used may considered as generalizable and worthy of publication. | Thank you for this accurate summary of the paper. |
| There are however, substantial problems with the methodology and what was done in reality: 1. mild head injury and (significant) traumatic brain injury were assessed together as one group. These are actually two different pathologies. | We take your point that treatment and management of mild and severe head injury are quite different, although in rare instances, an apparently mild head injury can mask a severe injury.  To differentiate between appropriate care for children with mild, and appropriate care for children with severe head injury, some of the indictors defined the group of patients to which they were aimed (e.g., *Children who presented with a moderate to severe head injury (GCS 3-13) …).* |
| 2. It seems to be there were few significant traumatic brain injury patients. Actually, in the presentation of the patients, only age and gender are provided. Data concerning the patients' degree of injury can only be extracted from the indicators (which is difficult). It seems from these indicators that around 16 had GCS of 9-13 and 31 had GCS of 3-13… In this sense, the authors need to describe how many patient records with mild head injury and how many patients with significant traumatic brain injury did they evaluate per site. | This is true: there were fewer children with severe head injury (reflecting its lower incidence in the study population). Some children moved from moderate to mild over time as their GCS improved. GCS was not always documented. This makes it not possible to categorise the patients in this way. |
| Since the actual findings of the survey itself are mainly of local significance, the authors should also concentrate their discussion to issues concerning the methodology (beyond the idea of structured electronic documentation): 1. In what way can the survey be improved (if at all) | Thank you for this suggestion. We have added in a comment about how the HEAD 54 could have been clearer.  HEAD 54 measured compliance of separate things and may have been ambiguous: *Children with a minor/mild head injury (GCS 14-15) whose parents were provided with information on when to return to the ED if deterioration occurs, were discharged from the ED without a period of observation.*  We have added to p.19 line 319: “In hindsight, this indicator may have been more usefully split into two as adherence rates do not tell us which aspect was not met.” |
| 2. In what way can compliance be improved. | Due to the evidence of practice variation, our suggestions of local review and addressing practice is appropriate. We have added in a suggestion about clarity and usability of CPGs and the need for peak bodies to review relevant CPGs to make the standards of care easier to follow.  *P. 21, line 378: Thirdly, there may be a role for peak bodies to review CPGs around head injury in children for clarity, currency and usability in the clinical setting.*  and note in the limitations section:  *P.20, line 339: Issues around the nature of CPGs may have been influential. As discussed in the main results paper of the CTK program^11^, low adherence and practice variation may reflect the failure of CPGs to be effectively structured, presented and implemented.*  We have already suggested improvements at the individual clinician, unit level and policy level – we have made these clearer and more explicit in the implications section (p.20) and added at P.18 Line 298*:*  *It does suggest that clinicians could improve the quality of their assessments by documenting these extra criteria and they are encouraged to consider adjusting their routine documentation to include them.*  (referring to presence/absence of seizures) |
